# Grouper *tshβ* Promoter-Driven Transgenic Zebrafish Marks Proximal Kidney Tubule Development

**DOI:** 10.1371/journal.pone.0097806

**Published:** 2014-06-06

**Authors:** Yang Wang, Zhi-Hui Sun, Li Zhou, Zhi Li, Jian-Fang Gui

**Affiliations:** State Key Laboratory of Freshwater Ecology and Biotechnology, Institute of Hydrobiology, Chinese Academy of Sciences, University of the Chinese Academy of Sciences, Wuhan, China; Leibniz Institute for Age Research - Fritz Lipmann Institute (FLI), Germany

## Abstract

Kidney tubule plays a critical role in recovering or secreting solutes, but the detailed morphogenesis remains unclear. Our previous studies have found that grouper *tshβ* (*gtshβ*) is also expressed in kidney, however, the distribution significance is still unknown. To understand the *gtshβ* role and kidney tubule morphogenesis, here, we have generated a transgenic zebrafish line *Tg(gtshβ:GFP)* with green fluorescent protein driven by the *gtshβ* promoter. Similar to the endogenous *tshβ* in zebrafish or in grouper, the *gtshβ* promoter-driven GFP is expressed in pituitary and kidney, and the developing details of proximal kidney tubule are marked in the transgenic zebrafish line. The *gfp* initially transcribes at 16 hours post fertilization (hpf) above the dorsal mesentery, and partially co-localizes with pronephric tubular markers *slc20a1a* and *cdh17*. Significantly, the GFP specifically localizes in proximal pronephric segments during embryogenesis and resides at kidney duct epithelium in adult fish. To test whether the *gtshβ* promoter-driven GFP may serve as a readout signal of the tubular development, we have treated the embryos with retinoic acid signaing (RA) reagents, in which exogenous RA addition results in a distal extension of the proximal segments, while RA inhibition induces a weakness and shortness of the proximal segments. Therefore, this transgenic line provides a useful tool for genetic or chemical analysis of kidney tubule.

## Introduction

Kidney is an organ that removes metabolic waste from the blood, and regulates the homeostasis of electrolytes and metabolite concentrations in a physiological range for supporting the functions of all other organs [1], [2]. In higher vertebrates, kidney is a complicated and highly branched system containing thousands of nephrons, the basic structural and functional unit of kidney, while in zebrafish, the simper kidney only has a bilaterally pair of nephrons [3]. Although there are some differences among various vertebrate kidneys, their cellular composition and molecular regulation are similar [4].

In zebrafish, pronephric tubule is also segmented into many regions similar to the segments in mammals. They include neck, proximal convoluted tubule (PCT), proximal straight tubule (PST), distal early (DE), corpuscle of Stannius (CS), and distal late (DL) [5]. Moreover, some factors influencing kidney segmentation have been identified in zebrafish [6]. For example, retinoic acid (RA) signaling has been revealed to play a principal role in proximal-distal segment determination of the pronephric tubule [5], [7], [8], [9], in which RA inhibits the distal segments and enables development of the proximal segments in a concentration-dependent manner [5]. In addition, canonical Wnt signaling and zinc finger transcription factor *osr1* have been found to be required for the formation of proximal pronephric tubule [10], [11], [12], [13], and noncanonical Wnt pathway has been demonstrated to regulate morphogenesis of the proximal and intermediate pronephros [14]. Recently, transcription factor Hnf1b is also confirmed to play a key role in nephron proximo-distal segmentation [15]. However, how these specific tubule segments are regionalized has remained unclear in zebrafish pronephros [3], [16].

Cell fate tracing and tissue-specific transgene technique provide powerful tools to study tissue development and organ formation [17], [18], [19]. In zebrafish, several transgenic lines with green fluorescent protein (GFP) have been constructed to visualize morphogenesis of the kidney organ. For example, the podocyte-specific transgenic zebrafish lines have been generated, in which GFP expression is driven by *wt1b* gene promoter [20] or *podocin* gene promoter [21]. In transgenic line *Tg*(*dβh:EGFP*), the *dopamine-β-hydroxylase* (*dβh*) promoter-driven EGFP is expressed in interrenal gland [22]. In *Na, K-ATPase alpha1A4:GFP* transgenic line, GFP is highly expressed in pronephric epithelia that power the ion-transport activities [23]. In *ret1*:GFP zebrafish line, the *ret* loci-driven GFP expression appears in the most distal pronephric duct [24]. Furthermore, the ET33-D10 zebrafish line with GFP expression marks the proximal movement of the pronephric tubule, but it displays relative high background and a wide GFP distribution [25]. So far, the specific transgenic line that clearly marks proximal pronephric tubule remains absent.

Thyroid-stimulating hormone (TSH) is a glycoprotein secreted by anterior pituitary [26]. Until recently, it has been thought solely to mediate thyroid development and production of thyroid hormones that are important for metabolism, growth and development [27], [28]. TSH consists of two subunits, the alpha and the beta subunit. The alpha subunit is nearly identical to all glycoprotein hormones, while the beta subunit is specific to each one. However, TSH receptors are expressed more ubiquitously, suggested by radio-ligand binding and mRNA analysis [29], [30]. Significantly, our previous studies in sex reversal groupers [31] have found that grouper *tshβ* (*gtshβ*) is also expressed in kidney in addition to its expression in pituitary [32]. These findings imply that some potential physiological functions of the hormone remain unknown. To explore these functions and thereby to understand kidney tubule morphogenesis, we have cloned the promoter of *gtshβ* and preliminarily analyzed its activity [33], but the marking values and potentials have been not investigated. In this study, we have generated a stable transgenic zebrafish line *Tg(gtshβ:GFP)* driven by the *gtshβ* promoter, revealed its marking ability to understand kidney tubule morphogenesis, and exploited its potentials for signaling regulation or chemical analysis of kidney tubule development.

## Materials and Methods

### Fish Maintenance

Zebrafish were maintained in the aquarium of Freshwater Ecology and Biotechnology Laboratory. Spawning, fertilization and embryo development were raised at 28.5°C as described previously [34]. The animal treatments for this research were approved by the Institute of Hydrobiology Institutional Animal Care and Use Committee (Approval ID: keshuizhuan 0829).

### Genome Walking

Genomic DNA was extracted from fin clips of groupers by a standard phenol-chloroform method, and the genomic walking PCR programs were referred to the manual proposal as described previously [35], [36]. The conserved synteny analysis was performed as described [37].

### Whole-mount *in situ* Hybridization and Immunofluorescence Staining

*In situ* hybridization and immunofluorescence staining were carried out as previously described [18], [38]. Riboprobes were made by using DIG RNA labeling kit (Roche) for the following genes, including *gfp*, *slc20a1a*, *wt1a*, *cdh17*, *vasa*, *mhc, pax2a, hnf1ba* and *hnf1bb* [20], [39], [40], [41], [42]. The WISH embryos were embedded using OCT (Sakura), and were sectioned for direct observation. Vasa antibody was used at 1∶200 as described [43].

### Confocal Microscopy and Fluorophotometry

For analyzing GFP expression, embryos were treated in PTU (sigma) from 16 hpf. At the indicated time, embryos were anesthetized with 30 mg/ml MS-222 (Sigma) for imaging using Leica sp2 confocal microscope or Leica MZ16FA stereomicroscope as described [44].

30 embryos were dechorionated and lysed using passive lysis buffer (promega). The supernatants were then collected for fluorophotometric scan (TECAN).

### Morpholino Oligonucleotides, Constructs and mRNA Injections

Morpholino oligonucleotides of *dead-end* (*dnd*) [45], *pax2a* [46] and *hnf1ba* [47] were obtained from Gene Tools. The RFP-CVLS chimera mRNA [48] was synthesized by cloning the PCR amplified fragment into the pCS2+ vector and by using Message Machine-Kit (Ambion). The microinjections were performed as described previously [49], [50].

### RA Treatments

Embryos at 10 hpf were incubated in 0.5 um ATRA (Sigma), 16 um DEAB (sigma) or DMSO (control) in E3 embryo media overnight, and fixed for *in situ* hybridization.

## Results

### Evolutionary Conservation of *tshβ* and the Neighbor Genes

**Figure 1:**
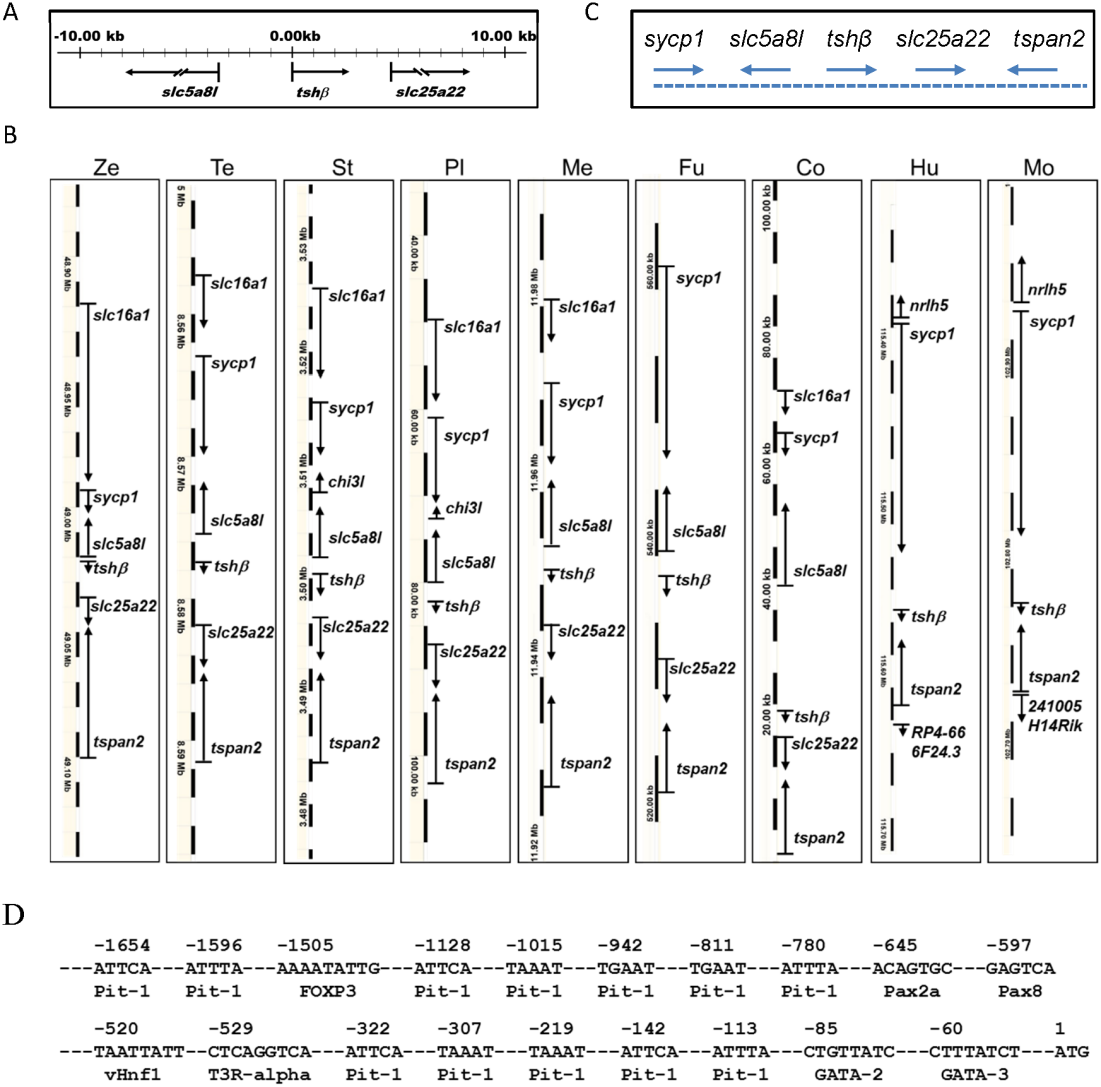
Genomic synteny conservation of *tshβ* and the neighbor genes. (A) The neighbor genes of *tshβ* in grouper. (B) Schematic diagram of genomic regions around *tshβ* in vertebrates. Ze: *zebrafish*, Te: *tetraodon*, St: *stickleback*, Pl: *platyfish*, Me: *medaka* Fu: *fugu*, Co: cod, Hu: *human* and Mo: *mouse*. (C) A representative diagram of the conserved genomic structure around *tshβ* in fish. (D) Putative pituitary and kidney-specific regulatory elements identified in the 1828 bp of proximal 5′ flanking regulation region in the grouper *tshβ* promoter.

Two neighborhood genes of grouper *tshβ* were firstly screened by genome walking. As shown in Figure 1A, the grouper *tshβ* localizes between *slc5a8l* (solute carrier family 5 (iodide transporter), member 8-like) and *slc25a22* (mitochondrial glutamate carrier 1), and both of them belong to the family of solute carrier. Subsequently, we compared the detailed genome regions around *tshβ* in vertebrates including zebrafish (*Danio rerio*), tetraodon (*Tetraodon nigroviridis)*, stickleback (*Gasterosteus aculeatus*), platyfish (*Xiphophorus maculatus*), medaka (*Oryzias latipes*), fugu (*Takifugu rubripes*), cod (*Gadus morhua*), human (*Homo sapiens*) and mouse (*Mus musculus*). Interestingly, the *tshβ* localization in genomes and the neighbor genes are almost identical in fish (Figure 1B), and a highly conserved synteny of *sycp1-slc5a8l-tshβ-slc25a22-tspan2* gene cluster (Figure 1C) exists in all of these fish. In mammals including human and mouse, *tshβ* also localizes in a *sycp1*-*tshβ*-*tspan2* cluster that is similar to that in fish on a large scale (Figure 1B).

### Characterization of *gtshβ* Promoter

Moreover, we analyzed potential regulatory DNA sequences in the upstream sequence of *gtshβ* start codon using PROMO program as described [51]. Some putative transcription factor binding elements important for mammalian pituitary development were found in this sequence. They include twelve consensus motifs for Pit-1 (at −1654, −1596, −1128, −1015, −942, −811, −780, −322, −307, −219, −142, and −113) [52], one for FOXP3 (at −1505) [53], one for T3R-alpha (at −529) [54], and one for GATA-2 (at −85) [55] respectively (Figure 1D). Interestingly, there are also many putative response elements critical for kidney morphogenesis in this region (Figure 1D). They include Pax2a responsive site (−645) [56], Pax8 responsive site (−587) [57], vHnf1 (also named as Hnf1b) responsive site (−520) [58], and GATA-3 responsive site (−60) [59], [60]. Therefore, the upstream region of *gtshβ* contains some significant information for directing its expression *in* *vivo*.

### Generation of Transgenic Zebrafish Line *Tg(gtshβ:GFP)*

A series of *gtshβ* 5′-flanking fragments with different length were cloned into the Tol2 vector [33]. These constructs were respectively injected into one-cell zebrafish embryos, and their activities were analyzed as described previously [17], [61]. Thereby, a proximal 1.8-kb DNA fragment was used to generate transgenic line. A total of 33 larvae (11%) with specific GFP signal were screened and raised to adulthood from the injected 300 embryos. By mating to wild type zebrafish, only 1 female F_1_ founder (3%) through germline transmission was identified from the 33 adults, and 25 F_2_ offspring with positive GFP signal were obtained from the 310 fertilized eggs between the female founder and wild type male due to the mosaicism of transgene in germline cells. As the positive F_2_ females were mated with the positive F_2_ males, about 25% embryos appeared strongly green fluorescence, and thereby, a stable and homozygous *gtshβ* promoter-driven transgenic zebrafish line was established in F_3_. When the homozygous transgenic zebrafish line was out-crossed to wild type, about 50% of the offspring showed specific GFP expression, suggesting that only one single integration site might exist in the transgenic line. We nominated the homozygous *gtshβ* promoter-driven transgenic zebrafish line as *Tg(gtshβ:GFP)* for all subsequent works.

### Dynamic GFP Expression Pattern during Embryogenesis in *Tg(gtshβ:GFP)*

**Figure 2:**
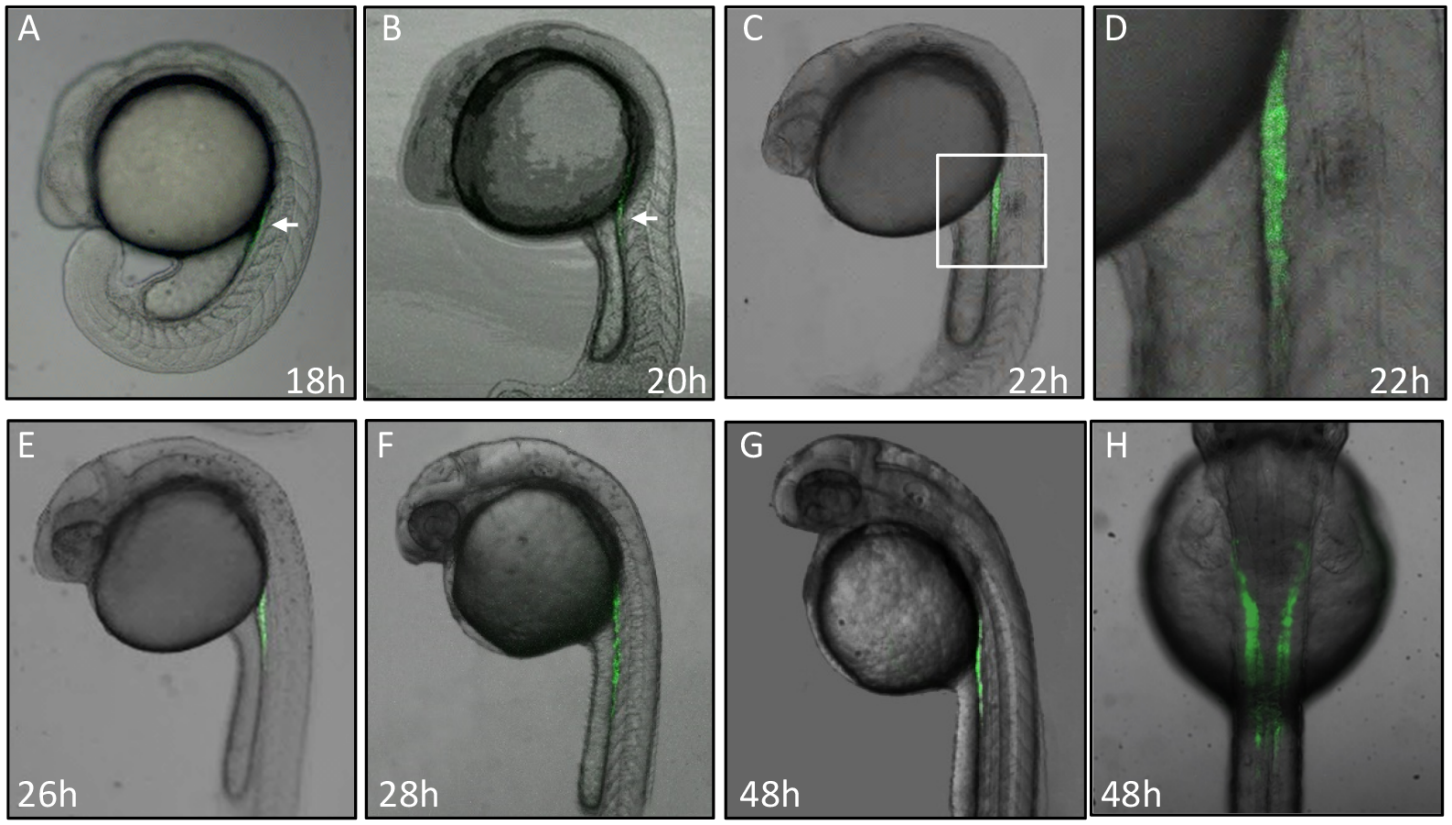
Dynamic GFP expression pattern during embryogenesis in *Tg(gtshβ::GFP)* line. (A, B, C, E, F and G) show the lateral view of *Tg(gtshβ:GFP)* embryos at the indicated stages. A dorsal view of 48 hpf embryo is shown in H. The arrows show the expressed GFP in A and B. The boxed area in C was shown with a higher magnification in D.

As judged by confocal microscopy, the GFP fluorescence signal is initially detectable from 18 hpf above the dorsal mesentery and persists throughout the subsequent embryogenesis in *Tg(gtshβ:GFP)* (Figure 2). The initial GFP signal localizes above the anterior region of the yolk extension, and extends from the adjacent fifth somite to the eighth somite (Figure 2A and 2B), where is the occurrence region of pronephros and gonad, although it is difficult to distinguish them at the early stages. As embryos develop, the GFP fluorescence becomes stronger and extends above the yolk sac, manifesting a bilateral tube-like structure (Figure 2C–2H). At 48 hpf, it displays a slight curve, which is likely the pronephric neck and tubule (Figure 2H). Therefore, the specific GFP expression driven by *gtshβ* promoter would be an excellent marker for pronephric tubule morphogenesis. In this paper, we only focused on the GFP expression in this region, although it can be detected in pituitary (data not shown).

### The *gtshβ* Promoter-driven GFP is Expressed in Pronephric Tubules

**Figure 3:**
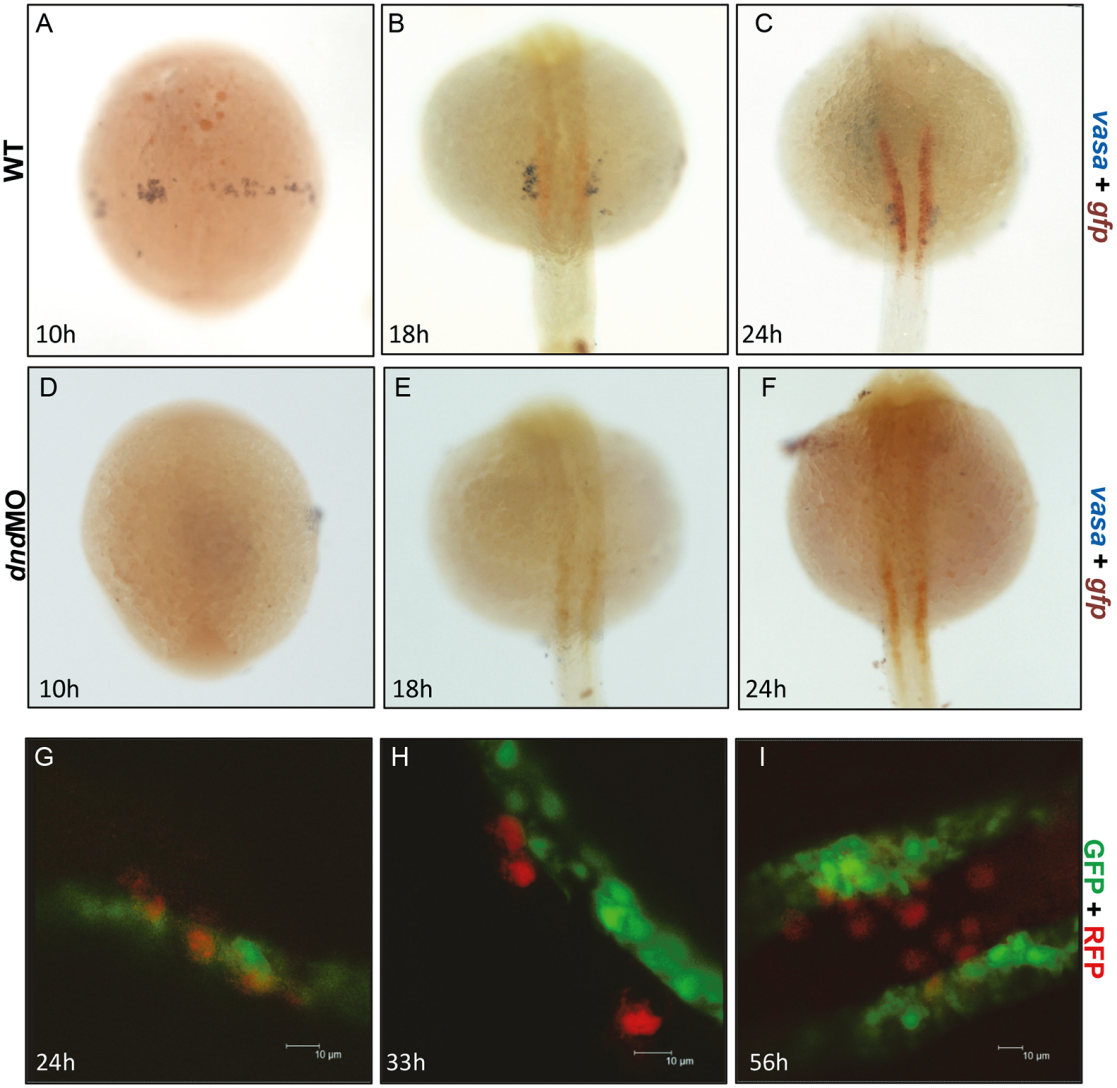
The *gtshβ* promoter-driven GFP was not localized with PGC. (A–C) dWISH of *gfp* (red) and *vasa* (purple) in *Tg(gtshβ::GFP)* embryos injecting control (A–C) and *dnd*-morpholino (*dnd*-MO) (D–F). (G–I) *Tg(gtshβ::GFP)* embryos injected with RFP-*nos* 3′UTR mRNA (red) were observed using confocal microscopy at the indicated stages. Bar = 10 um.

To reveal the exact localization of GFP driven by *gtshβ* promoter, we used double whole mount *in situ* hybridization (dWISH) to compare the transcript distribution between *vasa* (purple), a specific PGC marker, and *gfp* (red) in *Tg(gtshβ:GFP)* embryos. Similarly to the description reported previously [62], two clusters of the *vasa*-labeled PGCs were observed at the trunk border from somitogenesis beginning at 10 hpf (Figure 3A). At 18 hpf and 24 hpf, PGCs migrated and concentrated to the dorsal mesentery at the level of the yolk ball and yolk tube boundary (Figure 3B and 3C). At the corresponding stages, the red *gfp* transcript signal was found to localize on the bilateral strips in adjacent to and separated from the purple *vasa* signal (Figure 3A–3C). To confirm the above finding, the PGC-deleted embryos were produced via knocking down the *dnd* gene [45]. If *gfp* is transcribed in PGC, it would be hindered by PGC deletion. As shown in Figure 3D–3F, the red *gfp* transcript signal is still localized on the bilateral strips while the *vasa* expression is missing, indicating that the PGC deletion does not influence the expression of *gfp* message. We also applied RFP-*nos* 3′UTR to visualize PGCs by injecting the synthesized mRNA at one-cell stage as described [20], [63]. Similar to the results in dWISH assays (Figure 3A–3C), GFP was not co-localized with RFP in protein level and they were neighboring (Figure 3G–3I). Therefore, these data indicate that *gtshβ* promoter-driven GFP is not expressed in PGCs.

**Figure 4:**
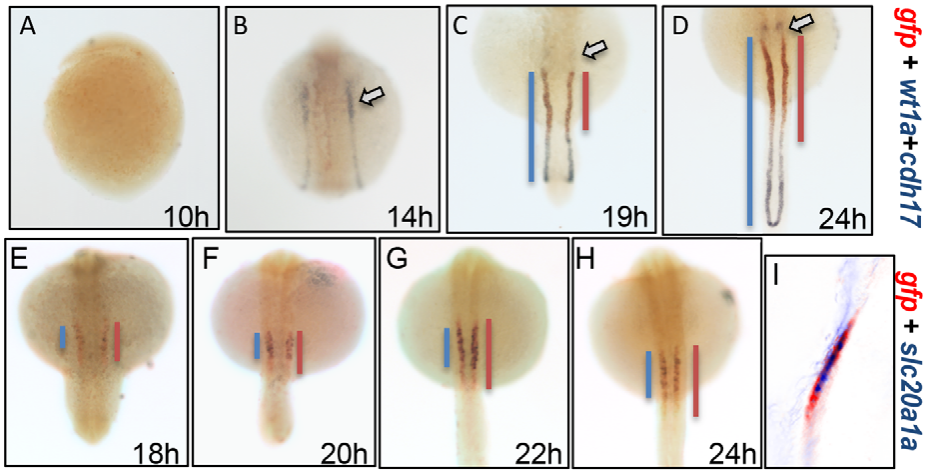
The *gtshβ* promoter-driven GFP is expressed in pronephric tubules. (A–D) dWISH analyses of *gfp* (red), *wt1a1* (purple) and *cdh17* (purple) in *Tg(gtshβ::GFP)* embryos at the somitogenic stages from 10 hpf to 24 hpf. (E–H) dWISH analyses of *gfp* (red) and *slc20a1a* (purple) in *Tg(gtshβ::GFP)* embryos at the somitogenic stages from 18 hpf to 24 hpf. (I) Nuance multispectral imaging of *gfp*-*slc20a1a*-double-stained *Tg(gtshβ::GFP)* embryos at 24 hpf. The podocytes stained with *wt1a1* are indicated by arrows. The blue or red lines are drawn according to the expression patterns of the kidney tubule markers.

To determine whether the GFP strips adjoining to PGCs belong to pronephros, we used pronephric markers to pursue the co-localization with *gfp* by dWISH. Wilms tumor gene *wt1a* is a transcription factor essential for the formation of glomerular structures [20]. Zebrafish *cdh17* is exclusively expressed in all pronephric tubules and ducts [40]. Therefore, the two markers combination could show the whole pronephros in zebrafish embryos (Figure 4A–4D) [64]. As shown in Figure 4, the *wt1a* and *cdh17* transcripts are partially covered by the *gfp* transcript at 19 hpf and 24 hpf (Figure 4C and 4D). Therefore, the overlapping region is likely the PCT segment that follows the Bowman’s capsule and neck segment. To test the deduction, a PCT segment marker *slc20a1a* was further applied to dWISH assay. Indeed, *slc20a1a* is co-localized with *gfp* in PCT segment from 18 hpf to 24 hpf (Figure 4E–4H). Interestingly, they are not totally overlapped, and the *slc20a1a* transcript resides in the anterior and middle parts of the *gfp* domain. Since Nuance multispectral imaging system could characterize all the spectral components in a sample [65], we further used it to clearly show the partial co-localization relationship between *slc20a1a* and *gfp* in pronephric tubule (Figure 4I). All together, these data indicate that the *gtshβ* promoter-driven GFP is mainly expressed in proximal pronephric tubules, and its distribution is more extended than PCT segment.

### Pax2a and Hnf1b Involve the *gtshβ* Promoter-driven Expression

**Figure 5:**
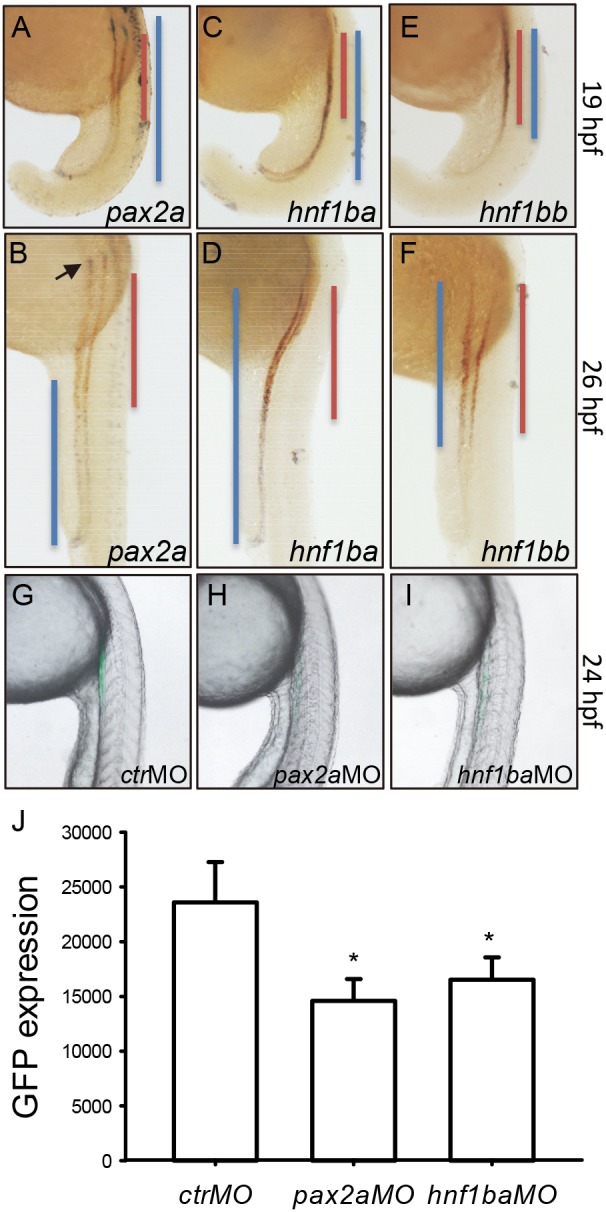
The *gtshβ* promoter-driven GFP is regulated by Pax2a and Hnf1b. (A–F) dWISH analyses of *gfp* (red) and kidney transcription factors (purple) as indicated in *Tg(gtshβ::GFP)* embryos at 19 hpf and 26 hpf. (G–I) show the lateral view of *Tg(gtshβ:GFP)* embryos injected with morpholinos as indicated at 24 hpf. (J) Fluorophotometric measurement of GFP in the embryos in G–I. The podocyte is indicated by arrow. The blue or red lines are drawn according to the expression patterns of the transcription factors and *gfp*, respectively. Error bars represent mean ± s.d., *P<0.001 one way AVOVA with Holm-Sidak method.

Some putative binding elements for Pax2a and Hnf1b, the known transcription factors for kidney development, were also identified in the *gtshβ* promoter region (Figure 1D). To test whether Pax2a and Hnf1b are required for the GFP expression in pronephric tubules, we firstly examined and compared their expression patterns with the *gtshβ* promoter-driven GFP in the *Tg(gtshβ:GFP)* embryos. As shown in Figure 5, the *pax2a* transcript expresses in the entire pronephros at 19 hpf, and co-localizes with *gfp* in the proximal tubules (Figure 5A). At 26 hpf, however, the *pax2a* transcript disappears in most of the *gfp* territory except a small part of posterior region (Figure 5B). In zebrafish, there are two paralogues of *hnf1b* (*hnf1ba* and *hnf1bb*), and both of them co-localize with *gfp* at 19 hpf and 26 hpf (Figure 5C–5F). The *hnf1ba* transcript is distributed in entire pronephric tubules (Figure 5C and 5D), whereas the *hnf1bb* distribution is shorter at the caudal area than that of *hnf1ba* (Figure 5E and 5F). Moreover, we analyzed the GFP expression in the Pax2a and Hnf1b knockdown *Tg(gtshβ:GFP)* embryos by using confocal imaging and fluorophotometry. As shown in Figure 5G–5J, the GFP expression level is significantly impaired by the Pax2a knockdown or Hnf1b knockdown. These results indicate that Pax2a and Hnf1b, the known transcription factors for kidney development, mediate the *gtshβ* promoter-driven expression.

### Onset Expression of *gtshβ* Promoter-driven *gfp* Transcript Occurs as the Formation of Pronephric Tubules

**Figure 6:**
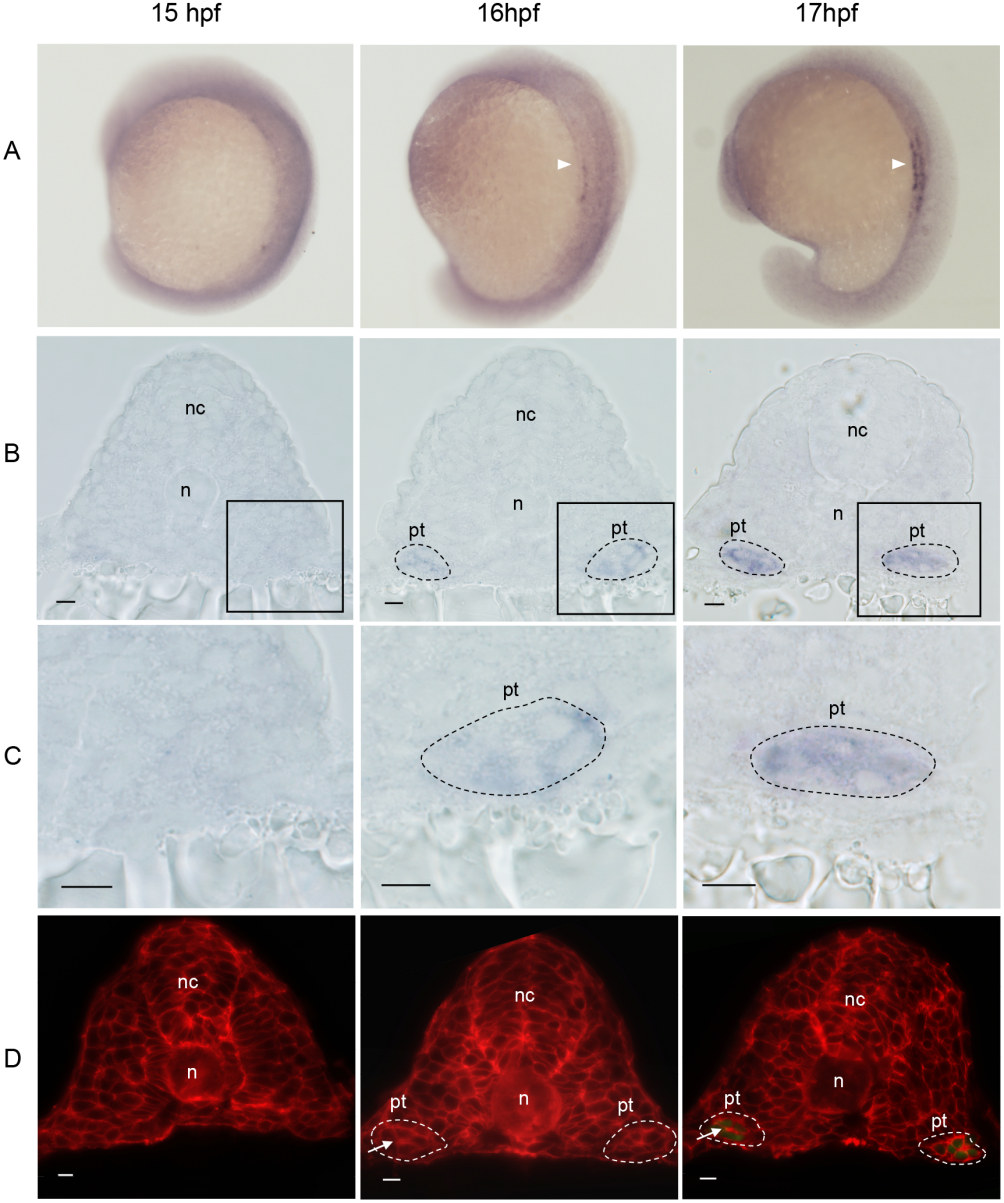
Onset expression of *gfp* mRNA during somitogenesis in *Tg(gtshβ:GFP)*. (A) WISH analysis of *gfp* (arrowheads) in 15, 16 and 17 hpf embryos, dorsal-lateral view. (B) Transverse sections of the embryos in A. (C) Higher magnification of boxed regions of B. (D) Cell boundary staining by the RFP-CVLS chimera protein on transverse sections of *Tg(gtshβ:GFP)* embryos. The *gfp*-positive cells are outlined by black dash lines. The tubular structures are outlined by white dash lines. Arrows indicate the lumen. nc: neural cord; n: notochord; pt: pronephric tubule. Bar = 10 µm.

To clarify the onset expression of *gfp* driven by *gtshβ* promoter, we examined *gfp* transcription in *Tg(gtshβ:GFP)* embryos at the early somitogenesis by using alkaline phosphatase-NBT/BCIP color system. The *gfp* mRNA is initially detected at 16 hpf when the embryos develop to 14-somite stage (Figure 6A). It is clear that the *gfp* transcription is earlier than the GFP protein translation at 18 hpf observed by using confocal microscopy (Figure 2). Furthermore, the *gfp* expressed cells form a clustered clump structure lateral to the both sides of somites (Figure 6B and 6C, black dash lines), and locate on the position where intermediate mesoderm (IM) develops to pronephros [66]. At 17 hpf (about 17-somite stage), the *gfp* signal becomes stronger (Figure 6A), and the cell clusters are more tightly organized than that at 16 hpf (Figure 6B and 6C, black dash lines). The data suggest that they might be undergoing reorganization events to form cylinder-type tubules structure during the stages.

To confirm the suggestion, we injected the RFP-CVLS chimera mRNA into 1-cell *Tg(gtshβ:GFP)* embryos to express a RFP that targets to the plasma membrane and thereby to visualize the tubular formation. As shown in Figure 6D, the limits of tubular structures begin to form at 16 hpf, and the cell boundaries indicated by the RFP-CVLS chimera protein obviously outline a characteristic circular shape at the 16-somite stage of 17 hpf. This implies that the cells in IM undergo structure organization to form a tubular tissue, and a small lumen can be observed in its middle (Figure 6D, arrows). Therefore, the onset expression of *tshβ* promoter-driven *gfp* occurs as the formation of pronephric tubules.

### The *gtshβ* Promoter-driven GFP Marks Pronephric Tubule Morphogenesis

**Figure 7:**
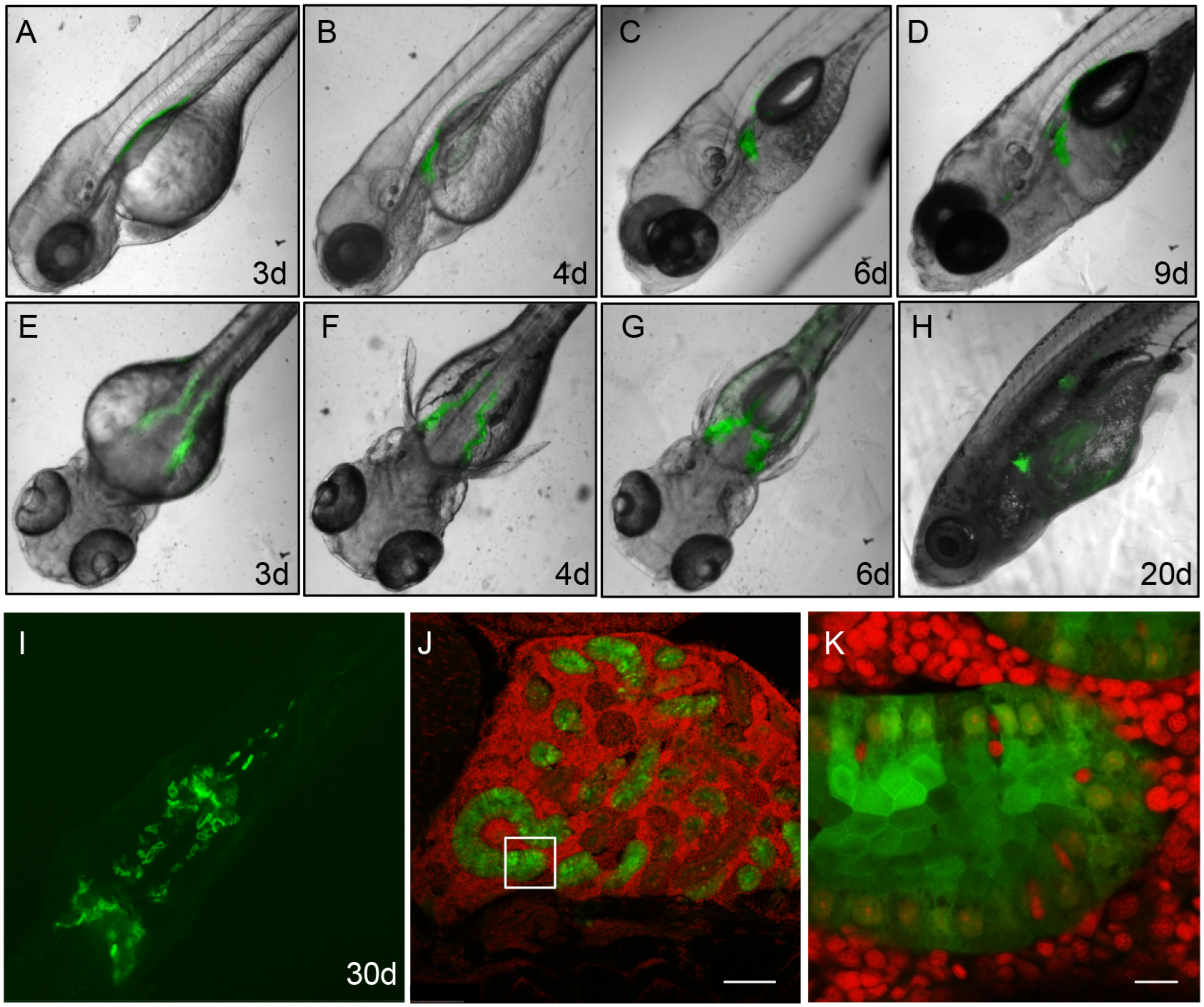
GFP expression pattern in *Tg(gtshβ::GFP)* larvae and adults. (A–I) show the lateral view (A, B, C, D and H) and the dorsal view (E–G, I) of *Tg(gtshβ::GFP)* larvae and adults from 3 dfp to 30 dfp. (J) The *Tg(gtshβ::GFP)* adult kidney section stained with DAPI (red). Bar = 100 µm (K) The higher magnification in the boxed area of J. Bar = 10 µm.

Subsequently, we examined pronephric tubule morphogenesis by pursuing the GFP expression pattern in the transgenic line from larvae to adults. As shown in Figure 7, GFP expressed in the hatched larvae appears in bilateral tubes of pronephros and persists into adults. The GFP expression domain moves forward and twists as the kidney develops (Figure 7A–7I). At 30 days after hatching, the cells expressing GFP clearly reside in a tubular structure (Figure 7J). They are squamous with cuboidal or columnar cell morphology and arrange very well (Figure 7K). Likely, these cells tightly contact to each other and form the epithelium tissue, which constitutes the nephric tubules [67], [68]. Therefore, the dynamic progression of nephron tubule morphogenesis throughout kidney development is marked by *gtsh*β promoter-driven GFP.

### The *gtshβ* Promoter-driven GFP Expression is Regulated by Retinoic Acid

**Figure 8:**
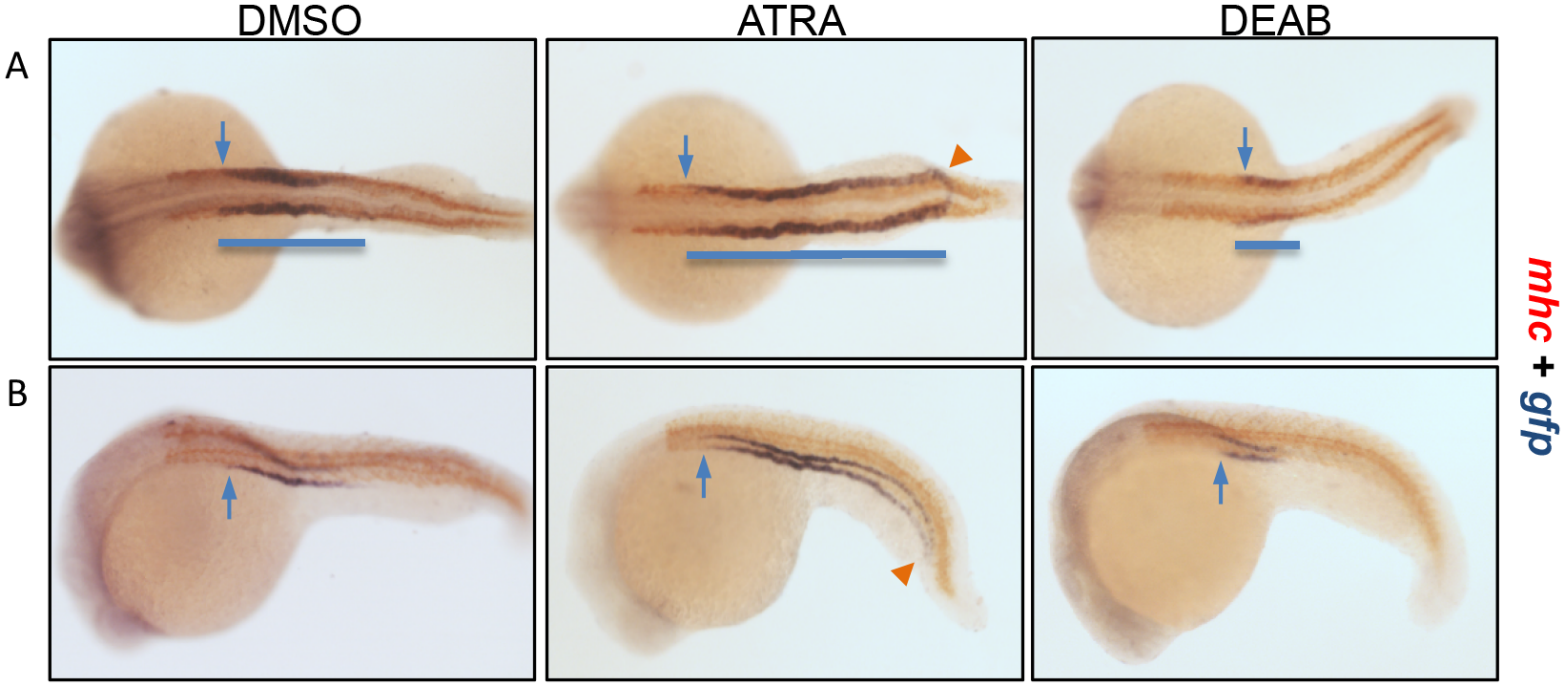
RA signaling regulates pronephric tubule segmentation. The *Tg(gtshβ::GFP)* embryos at bud stage were treated with DMSO (as control), ATRA or DEAB, and fixed at 22 hpf to perform double whole-mount *in situ* hybridization with *gfp* (purple) and *mhc* (red) probes. (A) Dorsal view. (B) Lateral view. Cloacas are indicated by arrowheads. The most anterior positions of *gfp* expression are indicated by arrows. The blue lines are drawn to mark the *gfp* expression patterns.

Retinoic acid (RA) signaling had been demonstrated to play a key function in controlling proximo-distal segmentation of the zebrafish pronephros [5]. Therefore, we used all-trans retinoic acid (ATRA), the ligand of RA signaling, and 4-diethylaminobenzaldehyde (DEAB), an effective inhibitor for the *de novo* RA synthesis to treat the *Tg(gtshβ::GFP)* embryos at bud stage, and thereby explored whether *gfp* expression is influenced by RA signaling. Moreover, to better define the changes of segment pattern, we mapped the expression domain of gfp relative to the somites with *myosin heavy chain* (*mhc*) [5]. As shown in Figure 8, the exogenous RA treatment results in distal expansion of the proximal segment, in which the *gfp*-positive strips are extended and fused at the prospective sites of cloaca (Figure 8A and 8B). In contrast, DEAB treatment attenuates and shortens the *gfp* expression pattern (Figure 8A and 8B). Interestingly, the front-end edge of *gfp* signal in DEAB-treated embryos moves posteriorly to the site of somite 5, compared with the somite 4 in DMSO controls (arrows in Figure 8). In the ATRA-treatment embryos, inversely, the leading edge of *gfp* expression shifts anteriorly to the somite 3 (arrows in Figure 8). These are similar to the previous report about the effects of RA on proximal tubule segmentation [5]. Therefore, the *gtshβ* promoter-driven GFP expression pattern in pronephros is regulated by retinoic acid, and RA signaling is required for the pronephric tubule residence and segment.

## Discussion

TSH is a glycoprotein hormone expressed and secreted from pituitary in mammals. It stimulates the production of thyroxine and then thiiodothyronine from thyroid to regulate the metabolism of almost every tissue. Besides a generally robust expression in pituitary, TSHβ mRNA was also found in gonads and kidney both in grouper [32] and zebrafish (data not shown). Genomic synteny analysis indicates that *tshβ* is localized with conserved genomic neighborhoods in many fish species (Figure 1A), suggesting the regulating elements may be similar in promoter region. Furthermore, our transgenic fish demonstrates that the promoter of grouper *tshβ* can direct the pituitary and kidney-specific expression of GFP in zebrafish, similar to the endogenous (Figure 1D). Therefore, the tissue distribution pattern of *tshβ* might be conserved in teleost fish. Although the significance of these expressions is unknown, the ubiquitous distribution of TSHR may suggest a divergent endocrine or paracrine TSH system in non-pituitary tissues [69], [70], [71].

Kidney tubule morphogenesis and segmentation are important for kidney function [72], but most of the process is unknown. In proximal tubule, phosphate, glucose, amino acid and bicarbonate are reabsorbed and transported. Only one transgenic fish line that marks the proximal tubule development was reported in zebrafish [25]. However, this transgenic fish line ET33-D10 displayed relative high background and a wide GFP distribution in various tissues, such as forebrain, hindbrain, spinal cord and ionocytes (http://plover.imcb.a-star.edu.sg/webpages/ET33-D10.html). In this case, the transgenic zebrafish line *Tg(gtshβ::GFP)* specifically expresses GFP in pronephric proximal tubule, besides in pituitary as previous report [73]. GFP is partially co-localized with proximal PCT segment marker *slc20a1a*, and the distribution pattern of GFP in pronephric tubule is a little extended than *slc20a1a* at the distal portion (Figure 4), which belongs to PST segment. When treated with RA, GFP signal elongates distally and almost connects at cloaca (Figure 8A), similar with the expression of the PST segment marker *trpm7* in response to RA [5]. Therefore, *gtshβ* promoter-driven GFP is specifically expressed in the proximal pronephric tubule, and is a valuable marker for PCT and PST segments.

Pax2a is one of the earliest acting transcription factors throughout the IM, and has been identified to control the mesenchyme-to-epithelial transition (MET) of nephron progenitors [74], [75]. Furthermore, Pax2a and the closely related Pax8 act upstream of *hnf1b* genes, which initiate the gene expression programs specific to pronephric segmentation [15], [76], [77], [78]. However, the epithelialization of IM is still unaffected in Hnf1b-deficient embryos, indicating that pronephric epithelialization and segmentation are separate and the early epithelialization is Hnf1b-independent [15]. The results suggest that both Pax2a and Hnf1b regulate the transcription of *gfp* through acing on *gtshβ* promoter (Figure 1D and 5). Consistently, *gfp* message is initially detected at 16 hpf, just as the formation of pronephric tubular lumen (Figure 6). It is the time that the EMT is undergoing and the segmentation is initialing. Therefore, GPF driven by the *gtshβ* promoter gives us a good marker to visualize the initial processes of pronephric development.

Through tracing the GFP signal, the *gtshβ* promoter-driven *Tg(gtshβ::GFP)* can be easily utilized to observe the convolution of the proximal tubules and the dynamic progression of nephron morphogenesis during the whole lifetime (Figure 2 and 7). Additionally, the expressed GFP can be used as a readout signal of the tubular development to detect the RA signaling alteration in response RA reagent treatment. Therefore, the transgenic line *Tg(gtshβ::GFP)* provides a potential tool for understanding morphogenesis and segmentation of pronephric tubules and for genetic or chemical analysis of kidney pathology [79].
